# Bromate reduction by *Shewanella* species depends on both endogenous and exogenous iron

**DOI:** 10.3389/fmicb.2025.1643578

**Published:** 2026-01-20

**Authors:** Natsuki Takahashi, Hiroko Fujiya, Haruhiko Sato, Shigeki Yamamura, Seigo Amachi

**Affiliations:** 1 Graduate School of Horticulture, Chiba University, Chiba, Japan; 2 Regional Environment Conservation Division, National Institute for Environmental Studies, Tsukuba, Japan

**Keywords:** bromate (BrO_3_^−^), *c*-type cytochrome, iron, redox mediator, reduction, *Shewanella*

## Abstract

In this study, we isolated *Shewanella* sp. strain M-Br, a bromate (BrO_3_^−^)-reducing bacterium, from seawater. In the presence of lactate as the electron donor, this strain reduced 250 μM bromate to bromide (Br^−^) in 3 days. Interestingly, bromate reduction by M-Br was ferric iron [Fe(III)]-dependent, and its bromate reduction rate was proportional to the Fe(III) concentration in the medium. Subsequently, a washed cell suspension of M-Br was prepared to determine whether the effect of Fe(III) on bromate reduction by M-Br is due to the endogenous iron sources, such as *c*-type cytochromes, or exogenous iron sources, such as redox mediators. Notably, cells pre-grown in the absence of Fe(III) did not reduce bromate, regardless of the addition of exogenous Fe(III). In contrast, cells pre-grown with Fe(III) reduced bromate upon the addition of exogenous Fe(III) to the cell suspension. Such iron-dependent bromate reduction was also observed in other *Shewanella* species, such as *S. putrefaciens* CN-32 and *S. oneidensis* MR-1. Our results suggest that both endogenous and exogenous iron are essential for bromate reduction by *Shewanella* species. Based on these results, we proposed a model, in which outer membrane multi-heme cytochromes, such as MtrC and OmcA, reduce Fe(III) to Fe(II), which further chemically reduces bromate to bromide. Such bacteria are useful for the removal of bromate, a potential human carcinogen, from drinking water.

## 1 Introduction

Bromate (BrO_3_^−^) is an ozonation byproduct of drinking water containing bromide ion (Br^−^) (Butler et al., 2005; Jahan et al., 2021). It is a potential human carcinogen, with a maximum contamination level of 10 μg L^−1^ in drinking water, as regulated by the International Agency for Research on Cancer (WHO, 2011). Optimization of the ozone level (Wert et al., 2007; Grefte et al., 2013), decrease in pH (Legube et al., 2004), and addition of ammonia (Neemann et al., 2004; Ikehata et al., 2013) and hydrogen peroxide (Wu et al., 2020) are efficient methods to minimize bromate formation during ozonation. However, complete prevention of bromate formation remains challenging. Various physical, chemical, and biological methods have been extensively studied for bromate removal from treated water (Jahan et al., 2021). Granular (Kirisits et al., 2000; Zhang et al., 2015) and powdered (Wang et al., 2010) activated carbon remove bromate via reduction and/or adsorption and are superior to chemical methods, such as addition of coagulants and reducing agents (Siddiqui et al., 1994; Gordon et al., 2002). However, use of granular and powdered activated carbon is not cost-effective. Therefore, biological methods not requiring any chemicals or energy for bromate removal have recently attracted considerable attention.

Biologically activated carbon (BAC) filters effectively remove bromate from drinking water, particularly after ozonation (Liu et al., 2012). Specifically, microorganisms, particularly bacteria, in the BAC filters reduce bromate to innocuous bromide (Lv et al., 2019). Moreover, dissolved oxygen level in the reactor should be kept low, and an appropriate carbon source should be added as an electron donor (Kirisits et al., 2001; Kirisits et al., 2002). To date, many studies have analyzed the microbial community structures in BAC filters and other bromate-reducing reactors (Kirisits et al., 2002; Assunção et al., 2011; Davidson et al., 2011; Liu et al., 2012; Luo et al., 2017; Zhong et al., 2018) and revealed that bromate-reducing bacteria are phylogenetically diverse, suggesting the ubiquitous distribution of bromate-reducing ability among different bacteria. However, only a few studies have attempted to isolate such bacteria from the reactors and natural environments (Hijnen et al., 1995; Davidson et al., 2011; Wang et al., 2022c).

We previously isolated the bromate-reducing bacterium, *Rhodococcus* sp. Br-6, from the soil (Tamai et al., 2016). This strain completely reduced 250 μM bromate in 4 days using the redox mediators, 2,6-dichroloindophenol (DCIP) and ferric iron [Fe(III)]. It first enzymatically reduced DCIP to DCIPH_2_, followed by the chemical reduction of Fe(III) to ferrous iron [Fe(II)], and finally Fe(II) chemically reduced bromate to bromide (Tamai et al., 2016). Although such hybrid biological and chemical bromate reduction mechanisms are interesting, the practical application of such systems is challenging because they require DCIP as a redox indicator. This difficulty motivated us to search for bacteria that can utilize iron alone as a redox mediator. In this study, we enriched bromate-reducing bacteria from seawater and isolated a new bromate-reducing bacterium, *Shewanella* sp. M-Br. Notably, bromate reduction by this strain was dependent on both the endogenous and exogenous iron. Additionally, our findings revealed the potential bromate reduction mechanisms of various *Shewanella* species, including the isolated strain.

## 2 Materials and methods

### 2.1 Enrichment of bromate-reducing Bacteria

Surface seawater was collected from Shin-Maiko Beach (Futsu, Chiba, Japan). An enrichment culture was prepared by inoculating 1 mL of seawater into 18 mL of minimal medium containing NH_4_Cl (0.535 g), KH_2_PO_4_ (0.136 g), MgCl_2_·6H_2_O (0.204 g), CaCl_2_·2H_2_O (0.147 g), NaCl (20 g), trace mineral element solution (5 mL), vitamin solution (10 mL), and NaHCO_3_ (2.52 g) per liter. The trace mineral element solution was based on that of DSM 318 medium (DSMZ, 1993), and the vitamin solution was based on that of DSM 141 medium (DSMZ, 1993). The minimal medium used in this study contained 25 μM of Fe(III) as FeCl_3_·6H_2_O instead of FeCl_2_·6H_2_O in the original DSM 318 medium. For anaerobic incubation, the minimal medium was dispensed into a 60-mL serum bottle under an N_2_/CO_2_ (80:20) gas stream. The bottle was sealed with a thick butyl rubber stopper and aluminum cap. After autoclaving at 121 °C for 20 min, sodium acetate and sodium bromate were added separately from sterile anaerobic stock solutions to achieve final concentrations of 20 mM and 250 μM, respectively. The final pH of the medium was 6.8–7.0. The bottles were subsequently incubated at 30 °C without shaking. Under microaerobic conditions, incubation was performed similarly to that under anaerobic conditions in a sealed serum bottle, but air substitution of the headspace and liquid phase with N_2_/CO_2_ gas was omitted. For microaerobic incubation, 0.174 g L^−1^ of K_2_HPO_4_ was added to the minimal medium, but NaHCO_3_ was excluded. The pH of the medium for microaerobic incubation was adjusted to 7.0 with NaOH. Under microaerobic conditions, oxygen in the medium is gradually consumed by bacteria, eventually resulting in anaerobic conditions (Tamai et al., 2016).

### 2.2 Isolation of bromate-reducing Bacteria

To isolate bromate-reducing bacteria, the enrichment culture sub-cultured five times under microaerobic conditions was serially diluted and spread on a solid minimal medium containing sodium acetate and 15 g L^−1^ of agar. In some cases, Luria–Bertani (LB) agar medium was used instead of a solid minimal medium. After aerobic incubation at 30 °C, bacterial colonies were randomly selected and purified. Subsequently, their bromate-reducing capacity was evaluated using a liquid minimal medium under microaerobic conditions. Specifically, 25 isolates were cultured with 250 μM bromate for 3 to 30 days under microaerobic conditions and bromate concentration in the medium was determined.

### 2.3 Identification and phylogenetic analysis of bacterial isolates

Genomic DNA of the isolated bacteria was extracted as previously described (Hiraishi, 1992). The 16S rRNA gene was amplified via polymerase chain reaction (PCR) using the bacterial consensus primers, 10F (5′-AGAGTTTGATCCTGGCTCAG-3′) and 1500R (5′-GGTTACCTTGTTACGACTT-3′). PCR products were purified using the QIAquick PCR Purification Kit (Qiagen, Hilden, Germany) and sequenced using the BigDye Terminator Cycle Sequencing Kit (Applied Biosystems, Foster City, CA, USA) and ABI Prism 3100 Genetic Analyzer (Applied Biosystems) using appropriate sequencing primers (Weisburg et al., 1991). The obtained 16S rRNA gene sequences were subjected to a Basic Local Alignment Search Tool search^1^ to determine the sequence similarity. The retrieved sequences were aligned using ClustalX version 2.0 (Larkin et al., 2007). Finally, a phylogenetic tree was constructed using the neighbor-joining method with the MEGA11 software package (Kumar et al., 2016).

### 2.4 Bromate reduction by the strain M-Br growing culture

Strain M-Br was first cultured aerobically in cultured in LB liquid medium. Cells were collected and washed twice with 20 mM Tris–HCl buffer (pH 7.0). The washed cells were then inoculated into a minimal medium containing 250 μM bromate, and cultured under anaerobic, microaerobic, and aerobic growth conditions. Sodium lactate was added as the electron donor and carbon source at a final concentration of 20 mM. Aerobic incubation was performed similarly to microaerobic incubation, except that the medium (20 mL) was dispensed into a 100-mL Erlenmeyer flask capped with a silicone plug, and the flask was incubated with shaking at 180 rpm.

To determine the effect of Fe(III) on bromate reduction, strain M-Br was grown in a minimal medium containing 0–100 μM FeCl_3_·6H_2_O under microaerobic conditions. To control the final Fe(III) concentration in the medium, Fe(III) in the trace mineral element solution was excluded from this experiment.

### 2.5 Bromate reduction by the washed cell suspensions of *Shewanella* species

*S. putrefaciens* CN-32 and *S. oneidensis* MR-1 were purchased from the American Type Culture Collection (ATCC BAA-453) and Japan Collection of Microorganisms (JCM 31522), respectively. Strain M-Br and these *Shewanella* spp. were grown in the minimum medium for 3 days under microaerobic conditions. The cells were collected, washed twice with 20 mM Tris–HCl buffer (pH 7.0), and resuspended in the same buffer to achieve an optical density at 600 nm of 0.5. Approximately 15 mL of the washed cell suspension was dispensed into a 100-mL Erlenmeyer flask or 60-mL serum bottle. Lactate, bromate, FeCl_3_·6H_2_O, and nitrilotriacetic acid (chelating reagent) were added to the suspension at final concentrations of 10 mM, 50 μM, 25 μM, and 335 μM, respectively. The flask was capped with a silicone plug and incubated with shaking at 180 rpm. In contrast, the serum bottle was degassed with N_2_ gas, sealed with a butyl rubber stopper and an aluminum cap, and anaerobically incubated without shaking.

To determine the effects of endogenous and exogenous Fe(III) on bromate reduction, washed cell suspensions were prepared from the cells grown with or without Fe(III) and supplemented with lactate, bromate, FeCl_3_·6H_2_O, and nitrilotriacetic acid, as described above. In some cases, FeCl_3_·6H_2_O was not added to the cell suspensions. Then, the cell suspensions were anaerobically incubated in serum bottles, as described above.

### 2.6 Bromate reduction by other Fe(III)-reducing Bacteria

To determine if other iron-reducing bacteria can reduce bromate, *Geobacter* sp. OR-1 (Ohtsuka et al., 2013) and *Anaeromyxobacter* sp. PSR-1 (Kudo et al., 2013) were used. They were grown anaerobically in the minimal medium containing 20 mM acetate and 20 mM Fe(III) as the electron donor and acceptor, respectively. Bromate was also added at a final concentration of 250 μM. In other cases, these strains were grown anaerobically with 20 mM acetate and 20 mM fumarate, which serves as an effective electron acceptor comparable to iron.

### 2.7 Analytical techniques

Bromate concentration was spectrophotometrically determined, as previously described (Tamai et al., 2016). Bromide was determined by the IC-2010 ion chromatography system (Tosoh, Tokyo, Japan) with the TSKgel SuperIC-Anion HR column (Tosoh) connected to the TSKgel SuperIC-A HS guard column (Tosoh). The mobile phase consisted of 2.2 mM NaHCO_3_ and 2.7 mM Na_2_CO_3_ at a flow rate of 1.0 mL min^−1^, and the column temperature was maintained at 40 °C.

## 3 Results

### 3.1 Enrichment and isolation of bromate-reducing Bacteria

Seawater was inoculated into a minimal medium and incubated with 250 μM bromate under anaerobic and microaerobic conditions (Supplementary Figure 1). Under anaerobic conditions, few bromate was reduced, even after incubation for 50 d. However, bromate was completely reduced in 14 d under microaerobic conditions. After five sub-cultures, bromate reduction was completed in 5 days. From this enrichment culture, multiple bacteria were randomly isolated and evaluated for their bromate-reducing capacity. Most isolated strains reduced bromate; however, their bromate reduction rates were very slow (data not shown). Among the tested strains, one exhibited the fastest bromate reduction rate, completely reducing 250 μM bromate in 4 days. This strain was selected as a novel bromate-reducing bacterium and designated as the strain M-Br.

Phylogenetic analysis via 16S rRNA gene sequencing revealed that the strain M-Br (NCBI accession number: LC900506) was closely related to the *S. putrefaciens* W3-18-1 and *S. putrefaciens* CN-32, with a sequence similarity of over 99% (Supplementary Figure 2). Other related bacteria included *S. oneidensis* MR-1, a representative dissimilatory Fe(III)-reducing bacterium.

### 3.2 Bromate reduction by strain M-Br under various growth conditions

**Figure 1:**
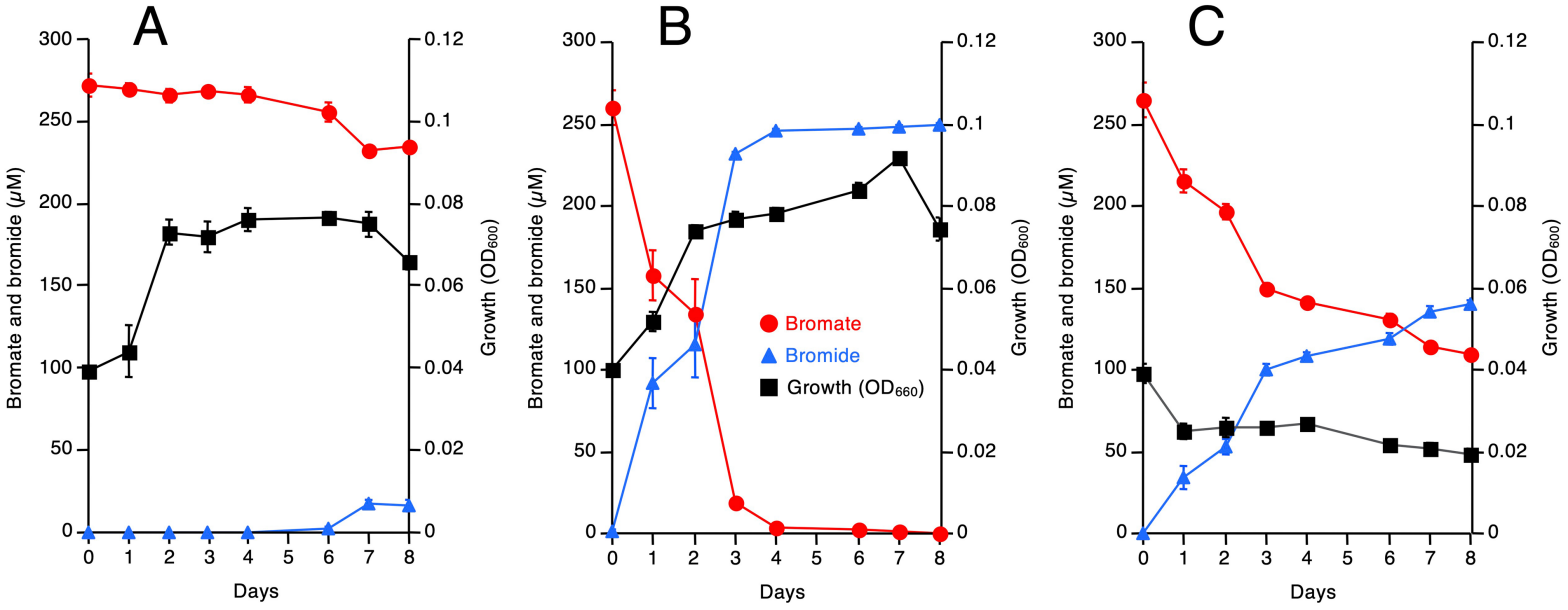
Growth, bromate reduction, and bromide production by strain M-Br grown under aerobic **(A)**, microaerobic **(B)**, and anaerobic **(C)** conditions in the minimal medium. Lactate was added as the electron donor and carbon source. All values are the mean values obtained for triplicate determinations, and bars indicate standard deviations. The absence of bars indicates that the error is smaller than the symbol.

M-Br was grown with bromate under aerobic, microaerobic, and anaerobic conditions, and its growth and bromate reduction rate were analyzed (Figure 1). Lactate was used instead of acetate as the electron donor and carbon source because it is generally the preferred substrate for the growth of *Shewanella* species under aerobic and anaerobic conditions (Zhang et al., 2021). As shown in Figure 1A, M-Br grew well but only reduced a small amount of bromate under aerobic conditions. Notably, M-Br grew better under microaerobic conditions than under aerobic conditions, completely reducing 250 μM bromate in 4 days (Figure 1B). Interestingly, the strain did not grow under anaerobic conditions, but it reduced approximately half of the bromate in 8 days (Figure 1C).

**Figure 2:**
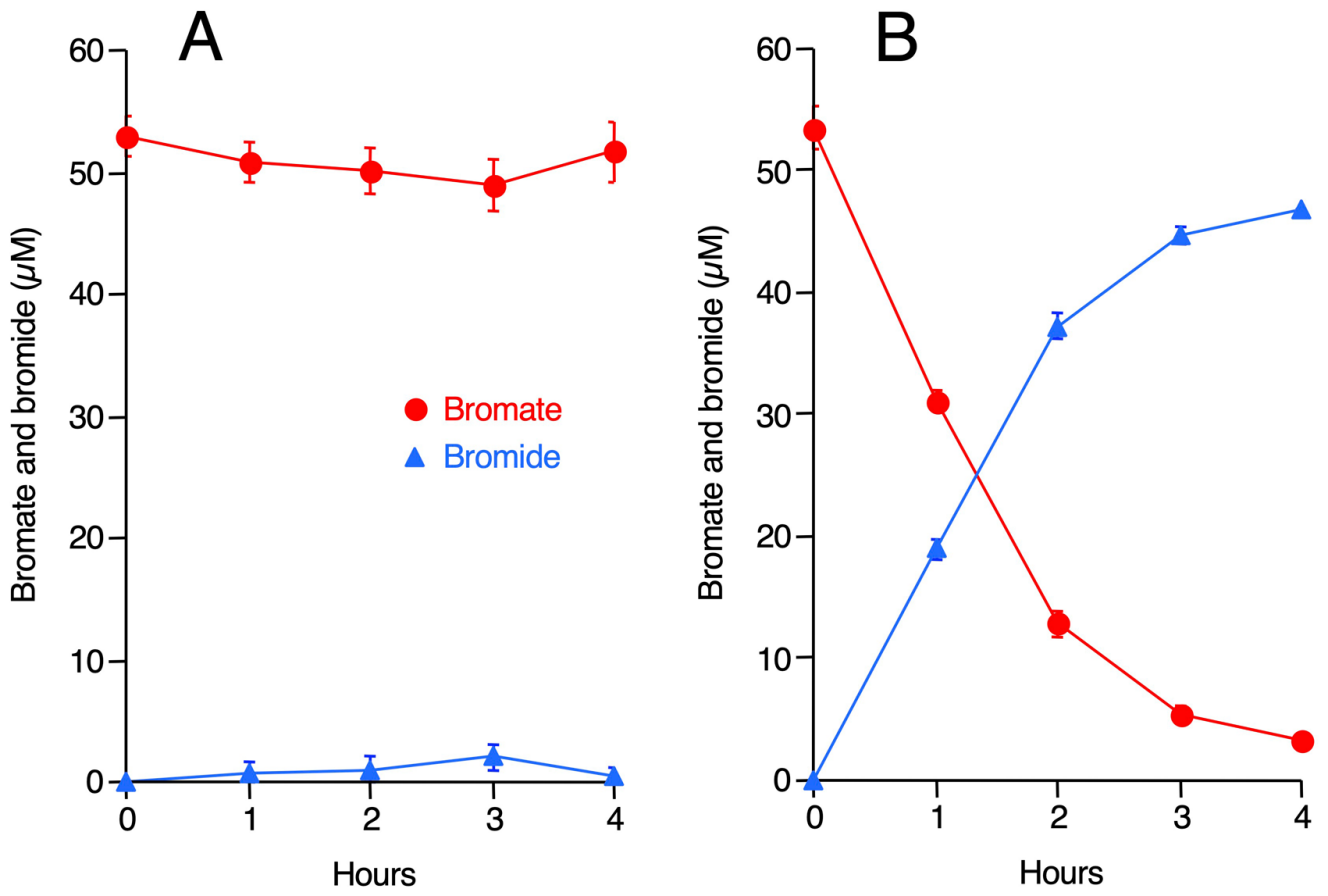
Bromate reduction and bromide production by washed cells of strain M-Br incubated under aerobic **(A)** and anaerobic **(B)** conditions. Lactate was added as the electron donor. All values are the mean values obtained for triplicate determinations, and bars indicate standard deviations. The absence of bars indicates that the error is smaller than the symbol.

Bromate reduction was much faster under microaerobic conditions than under aerobic and anaerobic conditions, suggesting that the strain M-Br reduces bromate preferably under oxygen-limited conditions but grows well under oxygen-rich conditions. To verify this hypothesis, a washed cell suspension of M-Br was prepared, and bromate reduction was examined under aerobic and anaerobic conditions (Figure 2). Indeed, no bromate was reduced under aerobic conditions, whereas 50 μM bromate was almost completely reduced in 3 h under anaerobic conditions.

### 3.3 Effect of Fe(III) on bromate reduction by strain M-Br

**Figure 3:**
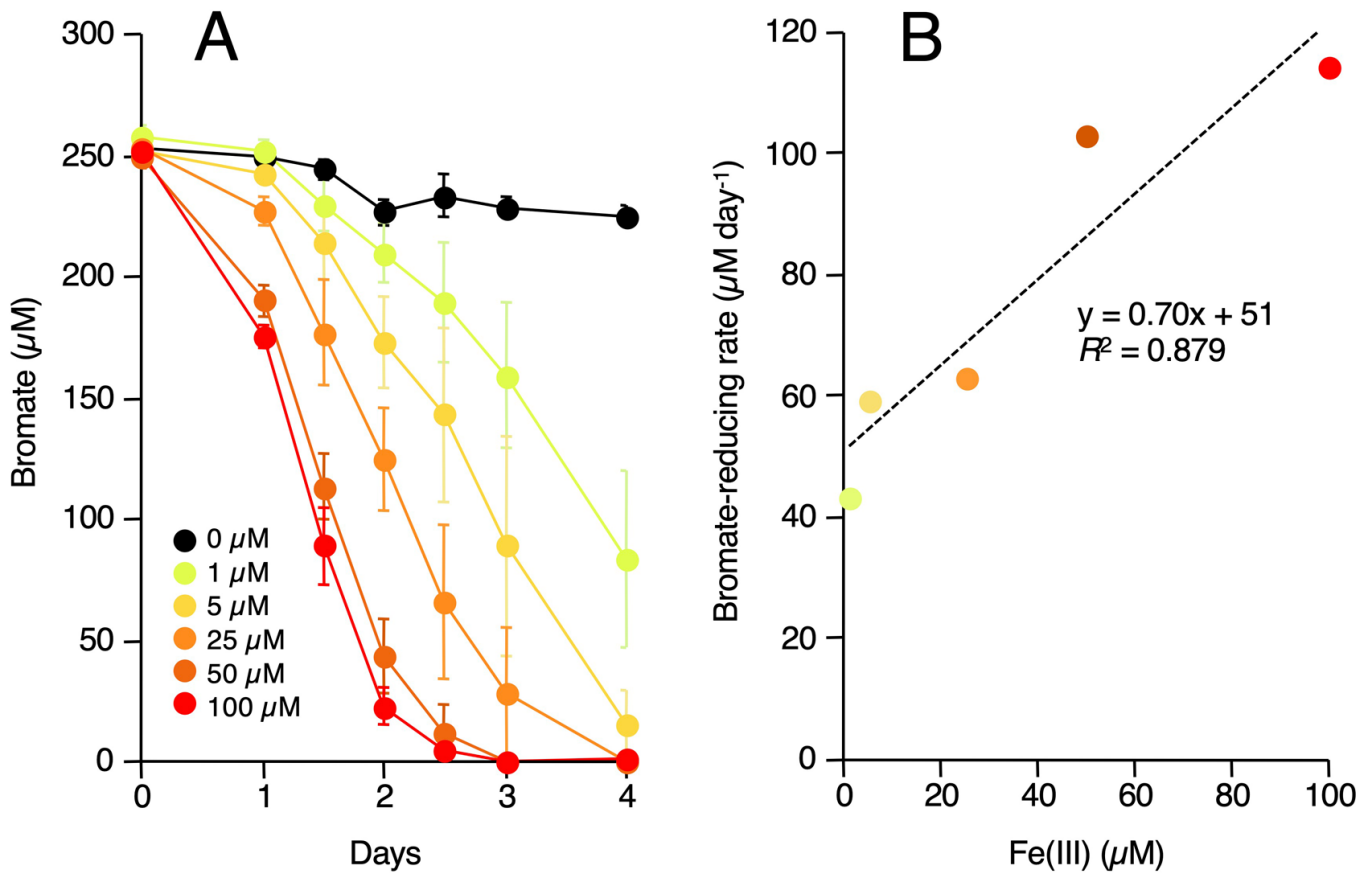
**(A)** Effect of ferric iron [Fe(III)] concentration in the medium on the bromate rection by strain M-Br. The strain was grown in the minimal medium containing 0 to 100 μM Fe(III). All values are the mean values obtained for triplicate determinations, and bars indicate standard deviations. The absence of bars indicates that the error is smaller than the symbol. **(B)** Relationship between Fe(III) concentration in the medium and bromate-reducing rates by strain M-Br. The bromate-reducing rates were calculated from the values of the day 2 (50 to 100 μM) or the day 4 (1 to 25 μM).

We previously reported that *Rhodococcus* sp. Br-6 uses Fe(III) as a redox mediator for bromate reduction (Tamai et al., 2016). As *Shewanella* species are well-known dissimilatory Fe(III)-reducing bacteria, strain M-Br possibly also uses Fe(III) as a redox mediator for bromate reduction. To verify this, a minimal medium lacking Fe(III) was prepared, and M-Br was grown with or without 1–100 μM Fe(III) under microaerobic conditions. As shown in Figure 3A, M-Br did not reduce bromate in the absence of Fe(III). In contrast, Fe(III) addition accelerated bromate reduction in a dose-dependent manner. As shown in Figure 3B, bromate reduction rate (μM day^−1^) of M-Br was positively correlated with the Fe(III) concentration in the medium.

### 3.4 Effect of Fe(III) on bromate reduction by washed cells of strain M-Br

**Figure 4:**
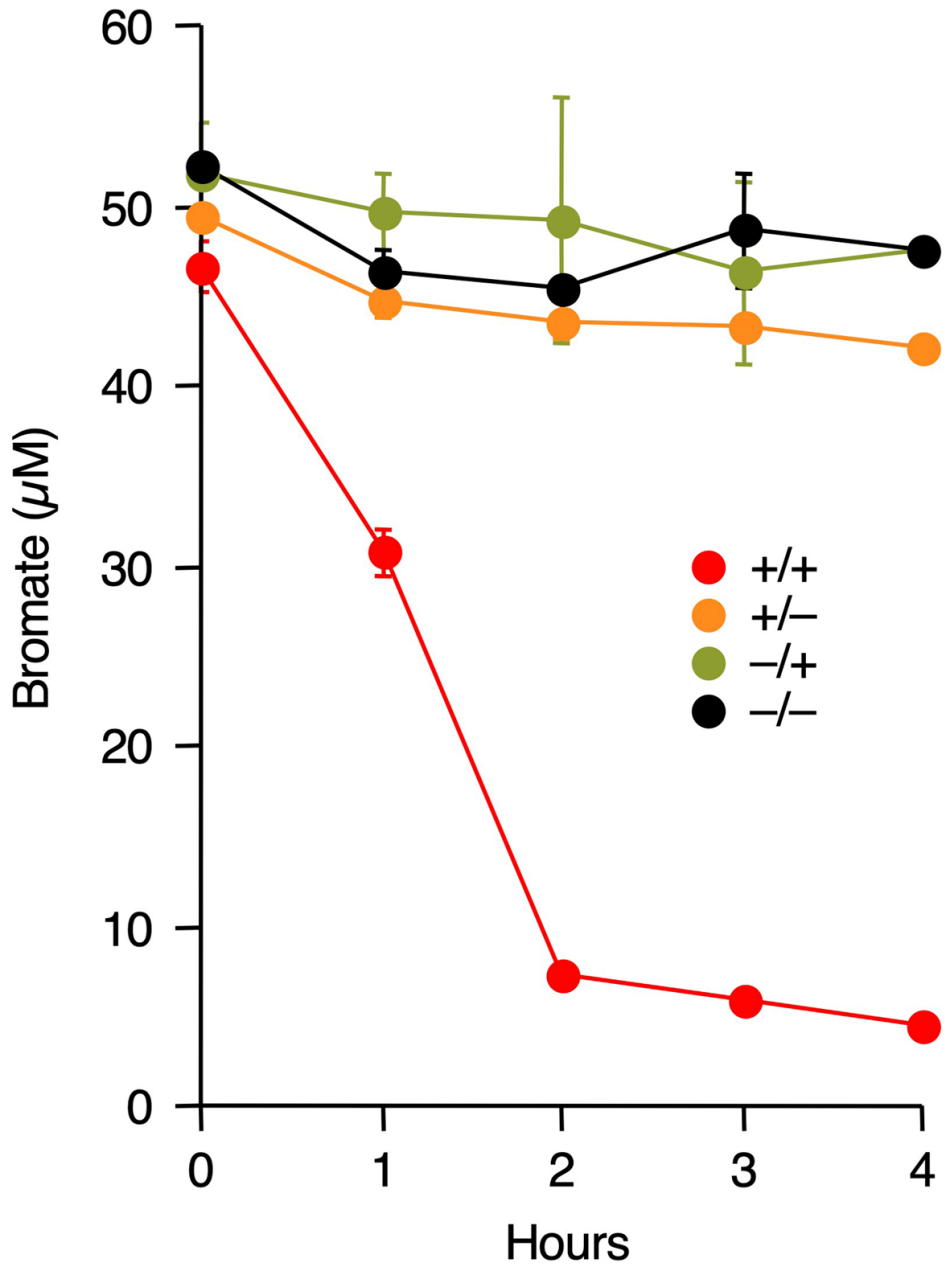
Effect of endogenous Fe(III) and exogenous Fe(III) on bromate reduction by washed cells of strain M-Br. The strain was pre-grown in the presence (red and orange circles) or absence (olive and black circles) of Fe(III). After washed cells were prepared, they were incubated with bromate in the presence (red and olive circles) or absence (orange and black circles) of exogenous Fe(III). All values are the mean values obtained for duplicate determinations, and bars indicate standard error of the mean. The absence of bars indicates that the error is smaller than the symbol.

Accelerated bromate reduction by Fe(III) is due to two possible reasons. First, Fe(III) functions as a redox mediator for bromate reduction, as in *Rhodococcus* sp. Br-6 (Tamai et al., 2016). Second, iron-associated proteins, such as *c*-type cytochromes, are involved in bromate reduction. To determine the specific mechanism, M-Br was pre-grown with or without Fe(III), and washed cells were prepared. The cells were incubated with bromate with or without exogenous Fe(III). As shown in Figure 4, bromate was reduced only when M-Br was pre-grown in the presence of Fe(III) and its washed cells were incubated with exogenous Fe(III). However, no significant bromate reduction was observed when M-Br was pre-grown in the absence of Fe(III) and its cells were incubated without exogenous Fe(III).

### 3.5 Effects of Fe(III) on bromate reduction by other *Shewanell*a species

**Figure 5:**
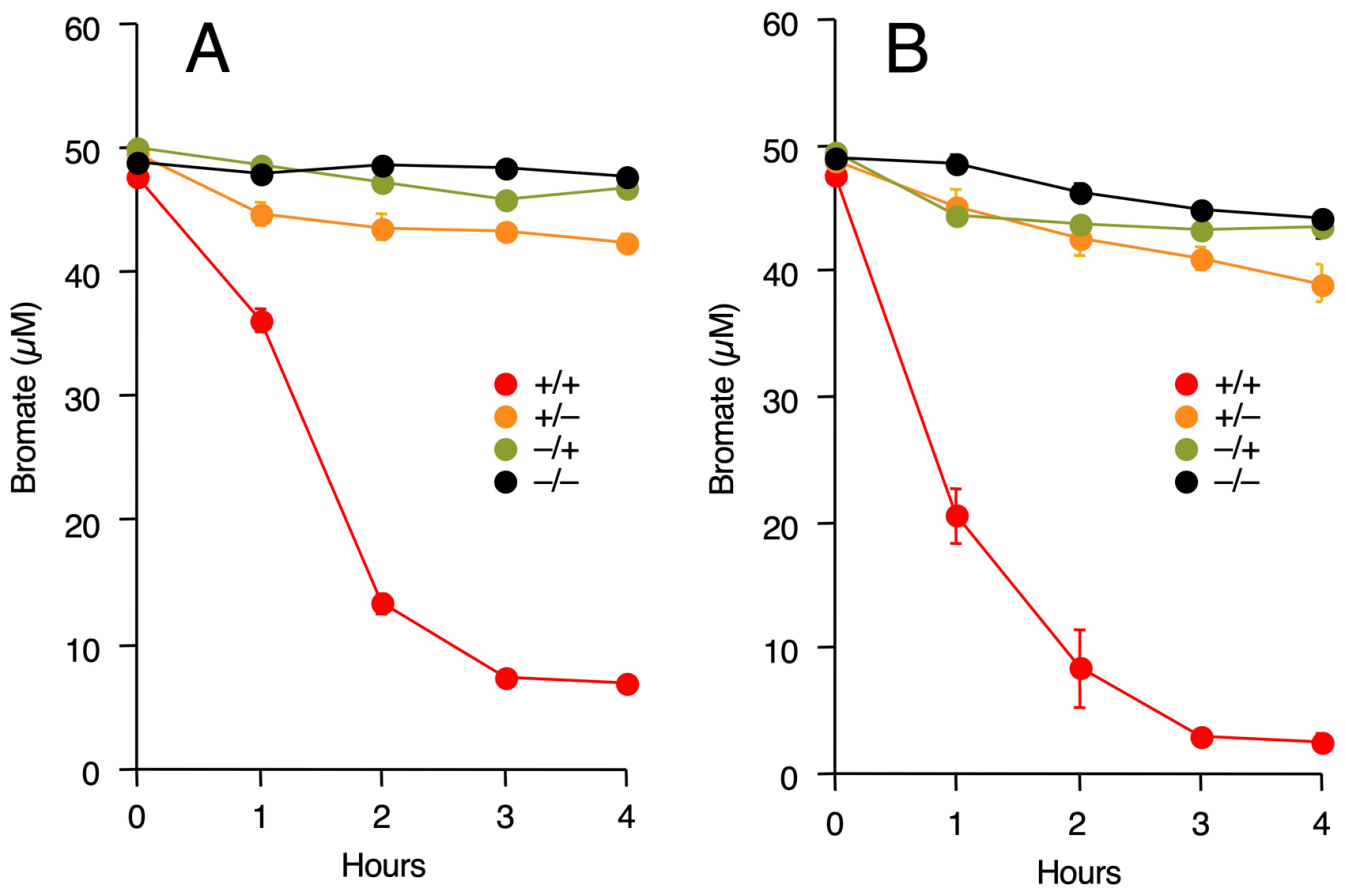
Effect of endogenous Fe(III) and exogenous Fe(III) on bromate reduction by washed cells of *Shewanella putrefaciens* CN-32 **(A)** and *S. oneidensis* MR-1 **(B)**. The strains were pre-grown in the presence (red and orange circles) or absence (olive and black circles) of Fe(III). After washed cells were prepared, they were incubated with bromate in the presence (red and olive circles) or absence (orange and black circles) of exogenous Fe(III). All values are the mean values obtained for duplicate determinations, and bars indicate standard error of the mean. The absence of bars indicates that the error is smaller than the symbol.

To determine whether Fe(III)-dependent bromate reduction is a general feature of *Shewanella* species, similar experiments were performed using *S. putrefaciens* CN-32 and *S. oneidensis* MR-1. As shown in Figure 5, bromate reduction by both bacteria was also Fe(III)-dependent, i.e., bromate reduction occurred only when the strains were pre-grown with Fe(III) and their washed cells were incubated with exogenous Fe(III).

### 3.6 Bromate reduction by other iron-reducing bacteria

**Figure 6:**
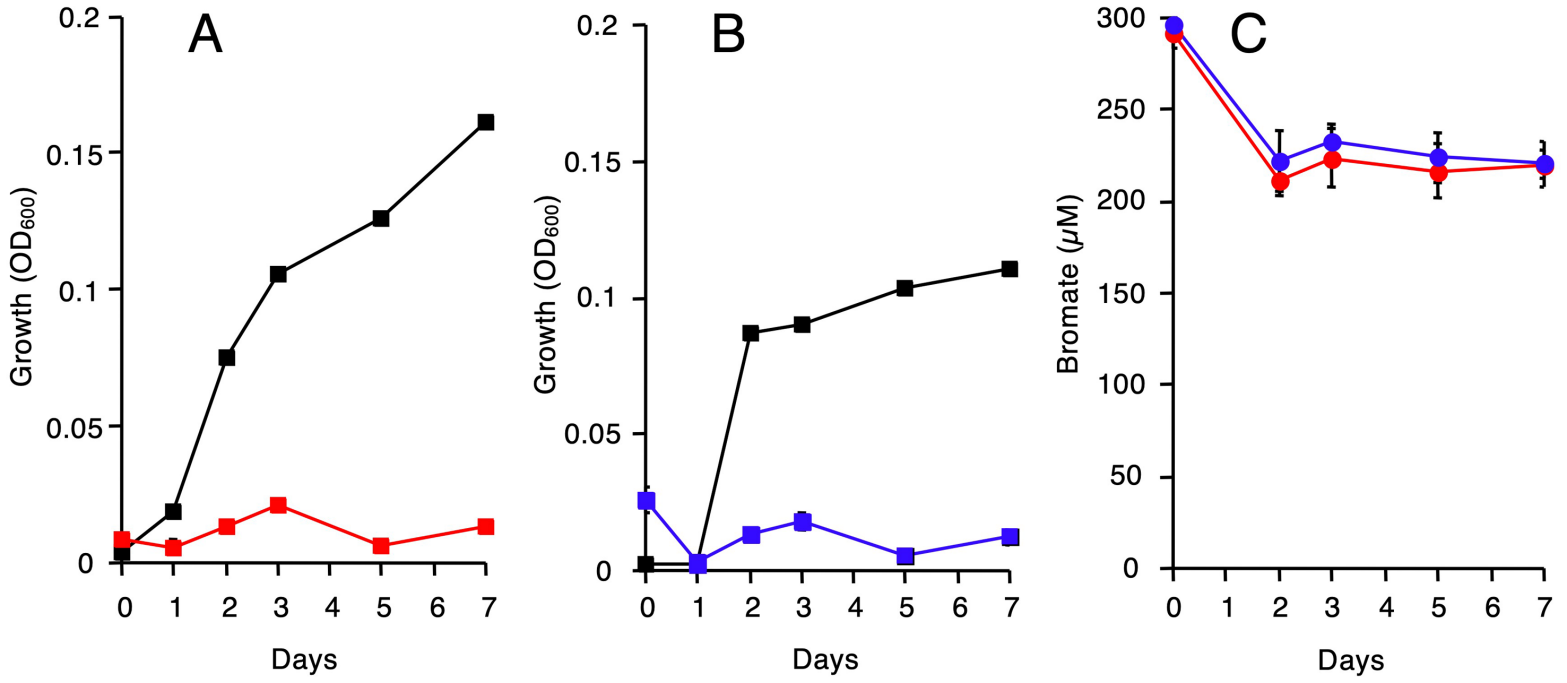
Growth and bromate reduction by other iron-reducing bacteria, *Geobacter* sp. OR-1 and *Anaeromyxobacter* sp. PSR-1. **(A)** *Geobacter* sp. OR-1 was grown with 20 mM fumarate as the electron acceptor in the absence (black squares) or presence (red squares) of 250 μM bromate. **(B)** *Anaeromyxobacter* sp. PSR-1 was grown with 20 mM fumarate as the electron acceptor in the absence (black squares) or presence (blue squares) of 250 μM bromate. **(C)** Bromate reduction by *Geobacter* sp. OR-1 (red circles) and *Anaeromyxobacter* sp. PSR-1 (blue circles). Growth conditions were same as **(A,B)**. All values are the mean values obtained for triplicate determinations, and bars indicate standard deviations. The absence of bars indicates that the error is smaller than the symbol.

To determine if other iron-reducing bacteria can reduce bromate, *Geobacter* sp. OR-1 (Ohtsuka et al., 2013) and *Anaeromyxobacter* sp. PSR-1 (Kudo et al., 2013) were grown anaerobically in the minimum medium containing 20 mM acetate and 20 mM Fe(III). Bromate was also added at a final concentration of 250 μM. However, due to the yellow color of the iron-containing culture supernatants, colorimetric quantification of bromate was difficult. In contrast, ion chromatographic analysis revealed that almost no bromide was produced (only up to 3 μM), indicating that bromate reduction had not proceeded. We next cultivated the strains anaerobically with 20 mM acetate and 20 mM fumarate [plus 25 μM Fe(III)], which serves as an effective electron acceptor comparable to iron. In the absence of bromate, both strains exhibited good growth, with OD₆₀₀ values ranging from 0.11 to 0.16 (Figures 6A,B). However, in the presence of 250 μM bromate, growth increased only up to a maximum OD₆₀₀ of 0.02. Quantification of bromate during this incubation showed that although a slight reduction of bromate occurred at the early stage of cultivation, no further reduction was observed thereafter (Figure 6C).

## 4 Discussion

In this study, we first attempted to isolate respiratory bromate-reducing bacteria using bromate as a terminal electron acceptor for growth. However, such bacteria were not enriched under anaerobic conditions (Supplementary Figure 1A). This is consistent with the results of our previous study, in which soil was incubated with bromate under anaerobic conditions (Tamai et al., 2016).

After enrichment under microaerobic conditions, we successfully isolated the bromate-reducing bacterium, *Shewanella* sp. M-Br. This strain grew well under aerobic and microaerobic conditions but reduced bromate only under anaerobic conditions (Figures 1, 2). Notably, M-Br did not grow anaerobically with bromate as the sole electron acceptor, indicating that bromate reduction by this strain is not a respiratory process (Figure 1C). The physiological reason why M-Br reduces bromate remains unclear. As bromate significantly inhibits the growth of specific bacteria, probably because of its strong oxidative power (Wang et al., 2022a), bromate reduction by M-Br and other bacteria is possibly a detoxification process.

Our results strongly suggest that bromate reduction by M-Br is dependent on both endogenous and exogenous iron (Figures 3, 4). M-Br cells grown without Fe(III) appeared white, whereas those grown with Fe(III) appeared pink to pale orange (data not shown). The latter color suggests the presence of heme in the cells, suggesting that endogenous iron is essential for cytochrome biosynthesis. In *S. oneidensis* MR-1, multi-heme *c*-type cytochromes, such as CymA, MtrA, MtrB, MtrC, and OmcA, are involved in the extracellular reduction of Fe(III) (Shi et al., 2012). Among these, CymA, MtrC, and OmcA catalyze the reduction of soluble Fe(III), such as chelated Fe(III) (Gescher et al., 2008; Shi et al., 2012). The draft genome sequence of M-Br (genome assembly number: ASM3624592v1) revealed the presence of orthologous genes encoding these proteins (Supplementary Figure 3). These findings suggest that M-Br reduces exogenous Fe(III) using multi-heme *c*-type cytochromes to form Fe(II), which chemically reduces bromate to bromide according to the following equation (Xie and Shang, 2005; Xie et al., 2008):


$${\mathrm{6Fe}}^{2+}+{{\mathrm{BrO}}_{3}}^{-}+{\mathrm{6H}}^{+}\to {\mathrm{6Fe}}^{3+}+{\mathrm{Br}}^{-}+{\mathrm{3H}}_{2}O.$$

Thus, exogenous iron may act as a redox mediator in bromate reduction, as previously reported in *Rhodococcus* sp. Br-6 (Tamai et al., 2016).

In our previous study, we observed the chemical reduction of bromate by Fe^2+^ (Tamai et al., 2016). Specifically, in the presence of six times the molar amount of Fe^2+^ relative to bromate, bromate was oxidized at a rate of 144 μM day^−1^. In the present study, the bromate reduction rate by *Shewanella* sp. M-Br was 114 μM day^−1^ (Figure 3), which agrees well with the above chemical reduction rate. In addition, M-Br reduced 250 μM bromate efficiently even in the presence of a stoichiometrically very low concentration of Fe^2+^ (Figure 3A). This observation indicates that the Fe^2+^ oxidized during bromate reduction was quickly re-reduced to Fe^2+^, suggesting that iron was functioning as a redox mediator in this process. Therefore, it seems reasonable to propose that iron functions not only in the biosynthesis of *c*-type cytochromes but also as a redox mediator.

Our results demonstrated that bromate reduction by *S. putrefaciens* CN-32 and *S. oneidensis* MR-1 was dependent on both endogenous and exogenous iron (Figure 5). Therefore, it is possible that bromate reduction by *Shewanella* species is generally iron-dependent. Recently, Wang et al. (2022a, 2022b) demonstrated bromate reduction by *S. decolorationis* Ni1-3 and *S. oneidensis* MR-1. The culture medium they used contained approximately 3 μM iron as FeSO_4_·7H_2_O, suggesting the involvement of iron in bromate reduction by the tested strains. Interestingly, Wang et al. (2022b) constructed gene deletion mutants of *S. oneidensis* MR-1 and found that CymA, MtrB, and MtrC are involved in bromate reduction. They proposed that these multi-heme cytochromes are necessary for the degradation of H_2_O_2_, which is possibly formed as a byproduct during bromate reduction. However, our results suggest that exogenous iron is required as a redox mediator for bromate reduction and that multi-heme cytochromes function as ferric [Fe(III)] reductases for the continuous supply of Fe(II) during bromate reduction. Additionally, Wang et al. (2022b) proposed that dimethyl sulfoxide reductase encoded by *dmsA* is a bromate reductase, as the Δ*dmsA* mutant showed significantly decreased bromate reduction. However, M-Br, *S. putrefaciens* CN-32, and *S. decolorationis* Ni1-3 do not harbor *dmsA* in their genomes, suggesting that this gene is not always necessary for bromate reduction.

**Figure 7:**
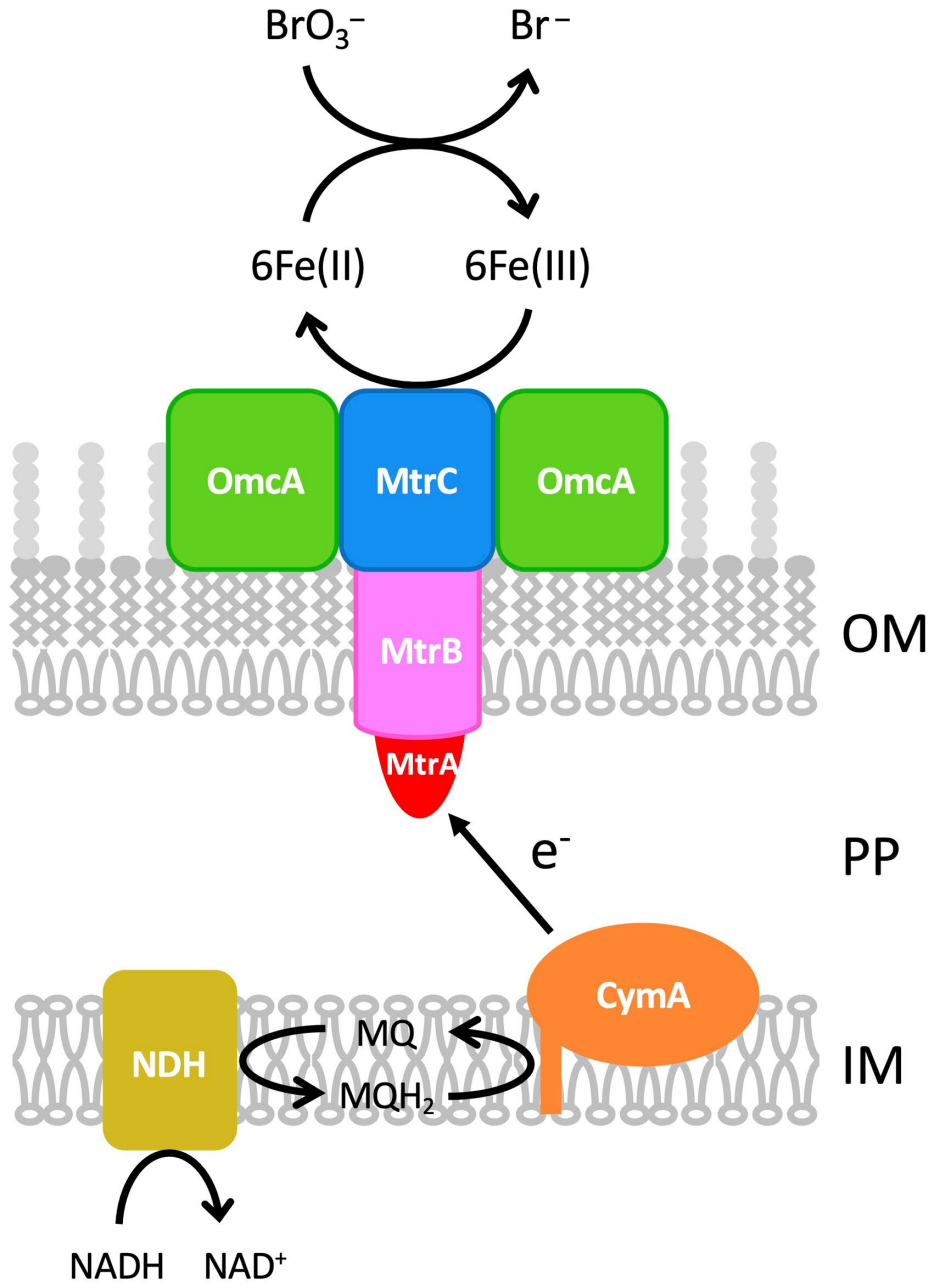
Proposed mechanism of bromate reduction by *Shewanella* species. Electrons derived from NADH is transferred to CymA via menaquinol (MQH_2_). CymA transfers the electrons to MtrA, MtrB, and MtrC, where Fe(III) is reduced to Fe(II) extracellularly. Finally, Fe(II) chemically reduces bromate to bromide at a molar ratio of 6:1. Fe(III) can also be reduced by OmcA and CymA. IM, Inner membrane; MQ, menaquinone; NDH, NADH dehydrogenase; OM, outer membrane; PP, periplasm.

Figure 7 shows a hypothetical model of bromate reduction by *Shewanella* species, including the strain M-Br. Electrons derived from NADH are transferred to menaquinone by NADH dehydrogenase, forming menaquinol in the inner membrane. CymA, a tetraheme *c*-type cytochrome (Myers and Myers, 1997), oxidizes menaquinol in the inner membrane and transfers the released electrons to MtrA in the outer membrane. MtrA is a decaheme *c*-type cytochrome embedded in MtrB, a porin-like protein (Shi et al., 2012). MtrAB facilitates electron transfer across the outer membrane to MtrC and OmcA on the cell surface. Both MtrC and OmcA are decaheme *c*-type cytochromes extracellularly reducing soluble Fe (III) to Fe(II) (Shi et al., 2006). Finally, Fe(II) chemically reduces bromate to bromide at a molar ratio of 6:1. Thus, bromate reduction by *Shewanella* species seems to be a hybrid process of biological and chemical reactions. However, this model is simpler than that of *Rhodococcus* sp. Br-6, in which DCIP and iron function as redox mediators (Tamai et al., 2016). According to this hypothetical model, one might suppose that all of iron-reducing bacteria can reduce bromate. We determined if iron-reducing bacteria maintained in our laboratory (*Geobacter* sp. OR-1 and *Anaeromyxobacter* sp. PSR-1) can reduce bromate in the presence of Fe(III), but neither growth nor bromate reduction was observed (Figure 6). This was probably because those strains were less tolerant to bromate. In other words, two key characteristics appear to be essential for a bacterium to function as a bromate-reducing organism: (1) tolerance to bromate, and (2) the ability to rapidly reduce small amounts of Fe^3+^. It is still unclear how bromate-reducing bacteria isolated to date reduce bromate, i.e., biologically, chemically, or through the hybrid process (Hijnen et al., 1995; Davidson et al., 2011; Wang et al., 2022a, 2022c).

In this study, the apparent bromate reduction rate of M-Br in the presence of 100 μM Fe(III) was 114 μM day^−1^ (Figure 3), which is the highest rate reported among bromate-reducing bacteria to date. For example, bromate reduction rate of the denitrifying bacterium, *Pseudomonas fluorescens* Br5, is 0.013–0.027 μM day^−1^ (Hijnen et al., 1995). Four bromate-reducing bacteria previously isolated from BAC filters or urban watershed exhibited bromate reduction rates < 0.04 μM day^−1^ (Davidson et al., 2011). *Rhodococcus* sp. Br-6 exhibits a high bromate reduction rate of 60 μM day^−1^, which is only approximately half of that of the strain M-Br (Tamai et al., 2016). Its superior bromate-reducing capacity over other bacteria highlights M-Br as a good candidate for the practical removal of bromate from treated waters. Although the addition of iron as a redox mediator is undesirable for water treatment, the amount of iron required for environmental levels of bromate reduction would be negligible. Future studies should explore nanomolar levels of bromate reduction by various *Shewanella* species for more valuable insights.

## Data Availability

The datasets presented in this study can be found in online repositories. The names of the repository/repositories and accession number(s) can be found in the article/Supplementary material.

## Footnotes

1
https://www.ncbi.nlm.nih.gov/BLAST/
