# Supplementary material for: Bromate reduction by Shewanella species depends on both endogenous and exogenous iron
Source: Front Microbiol. 2026 Jan 20;16:1643578. doi: 10.3389/fmicb.2025.1643578 (PMC12865605; doi:10.3389/fmicb.2025.1643578)
Supplement: Supplementary file 1 [file Data_Sheet_1.pdf]

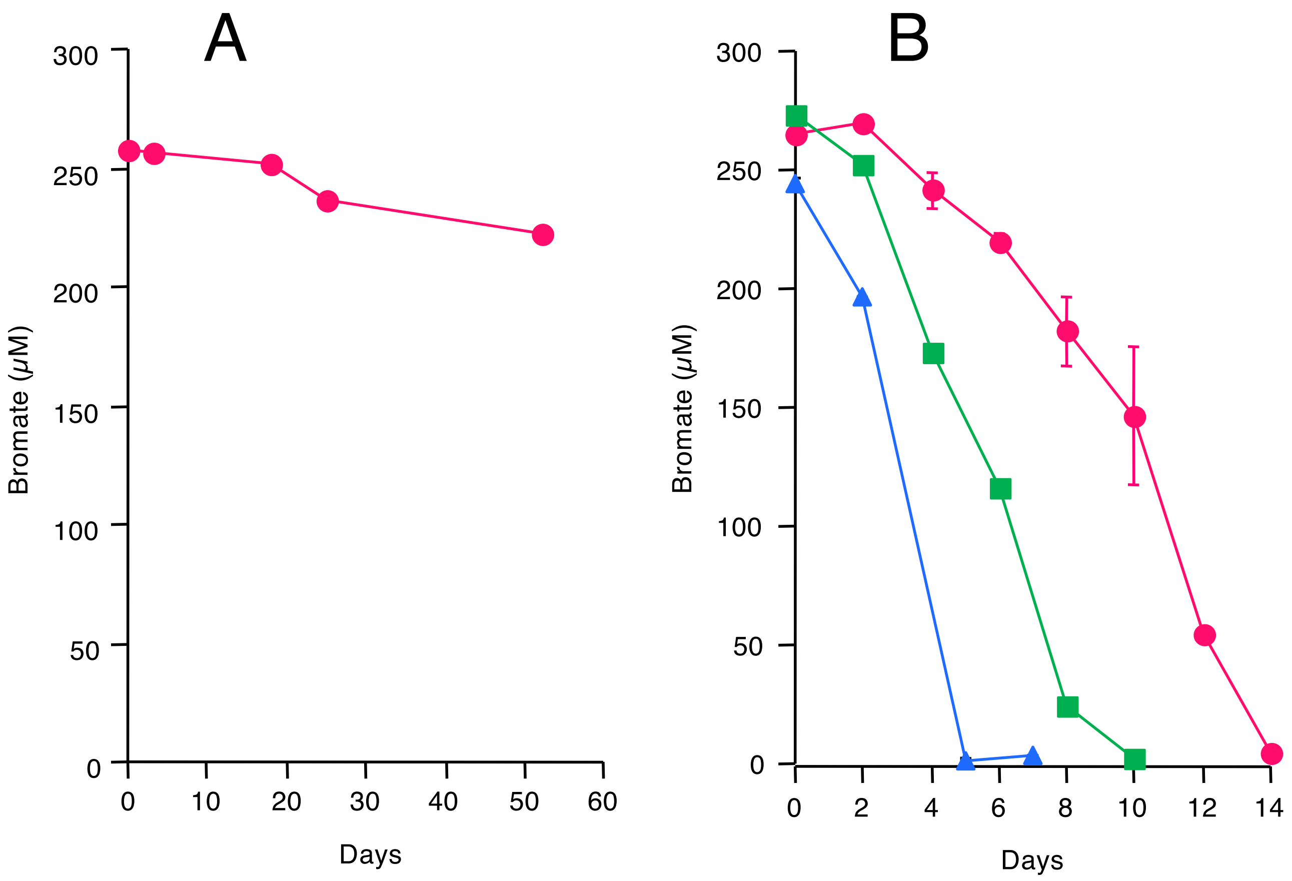

Fig. S1. Bromate reduction by enrichment culture incubated under anaerobically (A) or microaerobically (B). In the case of microaerobic incubation, bromate reduction after one time (green squares) and five times (blue triangles) of subculturing is also shown together with first enrichment (red circles). Acetate was added as the electron donor. All values are the mean values obtained for triplicate determinations, and bars indicate standard deviations. The absence of bars indicates that the error is smaller than the symbol.

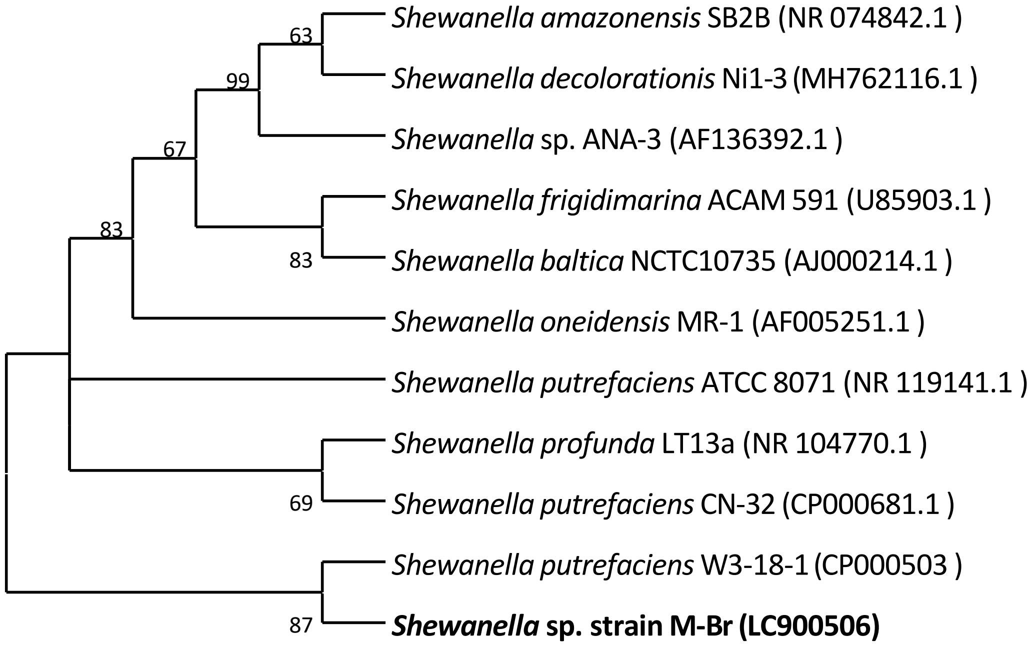

Fig. S2. Phylogenetic tree showing the relationship between strain M-Br and related bacteria within the genus *Shewanella*. The tree was constructed using the neighbor-joining method. Numbers at the branch nodes represent bootstrap percentages (1,000 replicates), and values < 50% are not shown.

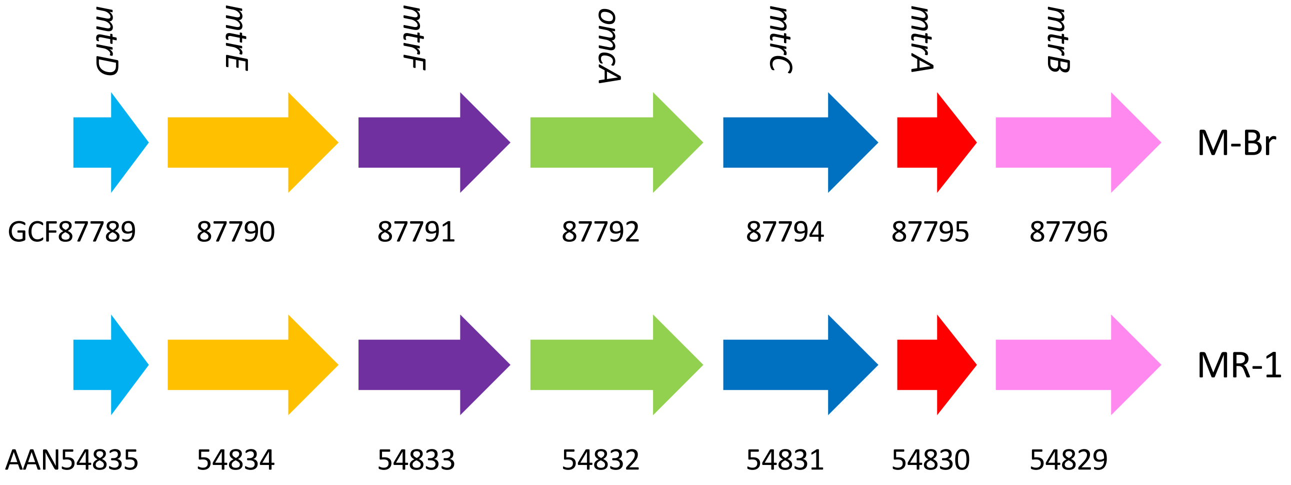

Fig. S3. The *mtr-omc* cluster in *Shewanella oneidensis* MR-1 and orthologous gene cluster in *Shewanella* sp. M-Br. The locus of each gene is also shown. The gene encoding CymA (GCF88689) is present in different region of M-Br genome.
